# The Role of Optimal Dyadic Mental Health in Couples Living With Lung Cancer: Interdependence, Congruence, or Balance?

**DOI:** 10.1093/geroni/igab046.1219

**Published:** 2021-12-17

**Authors:** Karen Lyons, Lyndsey Miller

**Affiliations:** 1 Boston College, Chestnut Hill, Massachusetts, United States; 2 School of Nursing, Oregon Health & Science University, Oregon, United States

## Abstract

Optimizing dyadic health is a central goal of dyadic frameworks. Yet, research has focused on interdependent individual health or the transactional nature of health within dyads. Emerging research has explored dyadic health through the lens of congruence and balance. This longitudinal study examined dyadic mental health in 76 couples (M = 67.88 ± 11.54) during the first year of lung cancer. As expected, multilevel modeling found mental and physical health of couples were significantly associated at baseline (p < .05). Congruence in mental health was significantly associated with changes in physical health over time for survivors (p < .05) but not partners, whereas balanced mental health had differential effects on the physical health of survivors and partners (p < .01). Discussion will focus on the implications of congruent versus balanced dyadic health for the couple, evaluation of interventions, and propose ways to define optimal dyadic health.

